# Dichloridobis{2-[(1*H*-1,2,4-triazol-1-yl)meth­yl]-1*H*-benzimidazole-κ*N* ^3^}­zinc(II)

**DOI:** 10.1107/S1600536813008283

**Published:** 2013-04-05

**Authors:** Wei-Peng Zhang, Jiao-Lin Zhang, Bao-Lian Hao, Huai-Xia Yang

**Affiliations:** a Pharmacy College, Henan University of Traditional Chinese Medicine, Zhengzhou 450008, People’s Republic of China

## Abstract

In the title complex, [ZnCl_2_(C_10_H_9_N_5_)_2_], the Zn^II^ ion is coordinated by two N atoms from two 2-[(1*H*-1,2,4-triazol-1-yl)meth­yl]-1*H*-benzimidazole (tmb) ligands and by two chloride ligands in a slightly distorted tetra­hedral geometry. In the tmb ligands, the benzimidazole rings systems are essentially planar, with maximum deviations from the mean plane of 0.021 (3) and 0.030 (3) Å, and form dihedral angles of 73.2 (2) and 83.5 (2)° with the triazole rings. In the crystal, N—H⋯N hydrogen bonds link complex mol­ecules into chains along [010]. In addition, weak C—H⋯Cl and C—H⋯N hydrogen bonds complete a three-dimensional network. Two weak intra­molecular C—H⋯Cl hydrogen bonds are also observed.

## Related literature

For background to complexes based on the 2-[(1*H*-1,2,4-triazol-1-yl)meth­yl]-1*H*-benzimidazole (tmb) ligand, see: Jin *et al.* (2012 ▶); Wang *et al.* (2012 ▶).
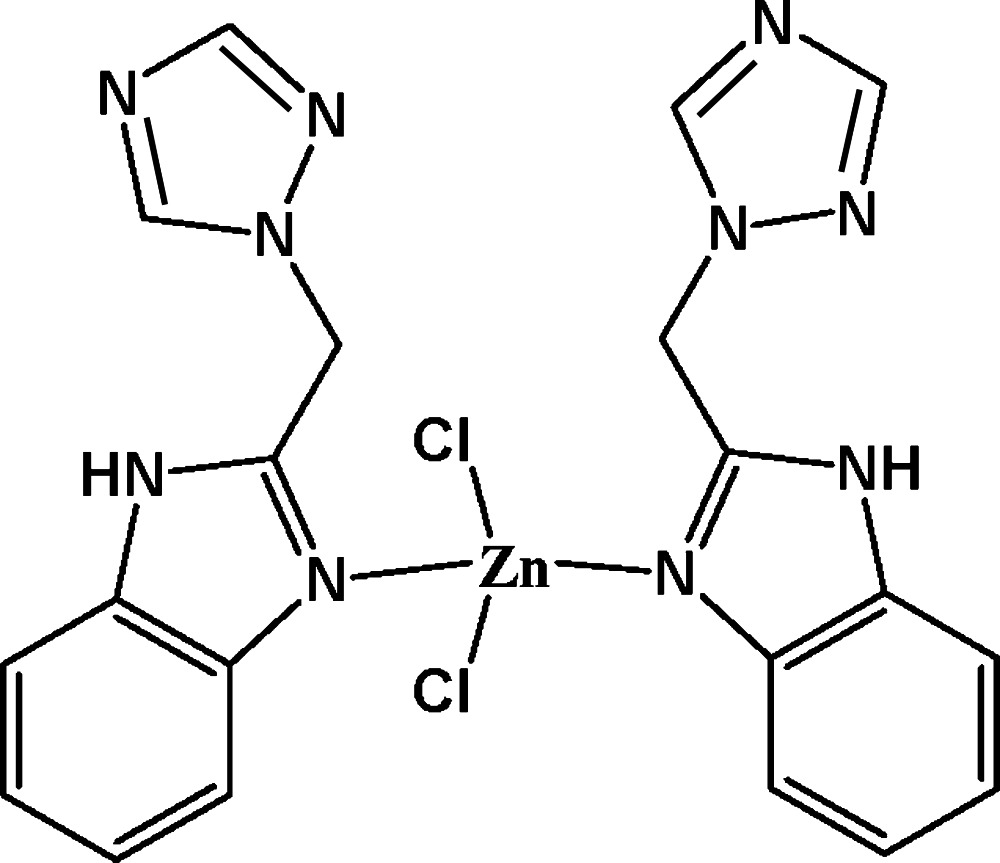


## Experimental

### 

#### Crystal data

[ZnCl_2_(C_10_H_9_N_5_)_2_]
*M* *_r_* = 534.73
Monoclinic, 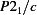

*a* = 11.571 (2) Å
*b* = 14.109 (3) Å
*c* = 16.357 (6) Å
β = 121.03 (2)°
*V* = 2288.2 (10) Å^3^
*Z* = 4
Mo *K*α radiation
μ = 1.34 mm^−1^
*T* = 293 K
0.18 × 0.17 × 0.08 mm

#### Data collection

Rigaku Saturn diffractometer
Absorption correction: multi-scan (*CrystalClear*; Rigaku/MSC, 2004 ▶) *T*_min_ = 0.795, *T*_max_ = 0.901
15780 measured reflections
4234 independent reflections
3396 reflections with *I* > 2σ(*I*)
*R*_int_ = 0.054

#### Refinement

*R*[*F* ^2^ > 2σ(*F* ^2^)] = 0.057
*wR*(*F* ^2^) = 0.108
*S* = 1.13
4234 reflections
298 parameters
H-atom parameters constrained
Δρ_max_ = 0.29 e Å^−3^
Δρ_min_ = −0.37 e Å^−3^

### 

Data collection: *CrystalClear* (Rigaku/MSC, 2004 ▶); cell refinement: *CrystalClear*; data reduction: *CrystalClear*; program(s) used to solve structure: *SHELXS97* (Sheldrick, 2008 ▶); program(s) used to refine structure: *SHELXL97* (Sheldrick, 2008 ▶); molecular graphics: *SHELXTL* (Sheldrick, 2008 ▶); software used to prepare material for publication: *publCIF* (Westrip, 2010 ▶).

## Supplementary Material

Crystal structure: contains datablock(s) global, I. DOI: 10.1107/S1600536813008283/lh5599sup1.cif

Structure factors: contains datablock(s) I. DOI: 10.1107/S1600536813008283/lh5599Isup2.hkl

Additional supplementary materials:  crystallographic information; 3D view; checkCIF report

## Figures and Tables

**Table 1:** Hydrogen-bond geometry (Å, °)

*D*—H⋯*A*	*D*—H	H⋯*A*	*D*⋯*A*	*D*—H⋯*A*
N2—H2*B*⋯N5^i^	0.86	2.04	2.899 (4)	177
N7—H7*B*⋯N10^ii^	0.86	1.96	2.814 (4)	172
C3—H3*B*⋯Cl1	0.97	2.83	3.641 (4)	142
C13—H13*B*⋯Cl1	0.97	2.73	3.628 (4)	154
C2—H2*A*⋯Cl1^iii^	0.93	2.77	3.596 (4)	148
C13—H13*A*⋯N4^iv^	0.97	2.62	3.261 (5)	124
C18—H18*A*⋯Cl1^v^	0.93	2.81	3.635 (4)	149

Symmetry codes: (i) 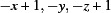; (ii) 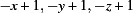; (iii) 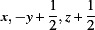; (iv) 
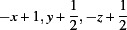
; (v) 
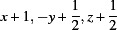
.

**Fig. 1.**
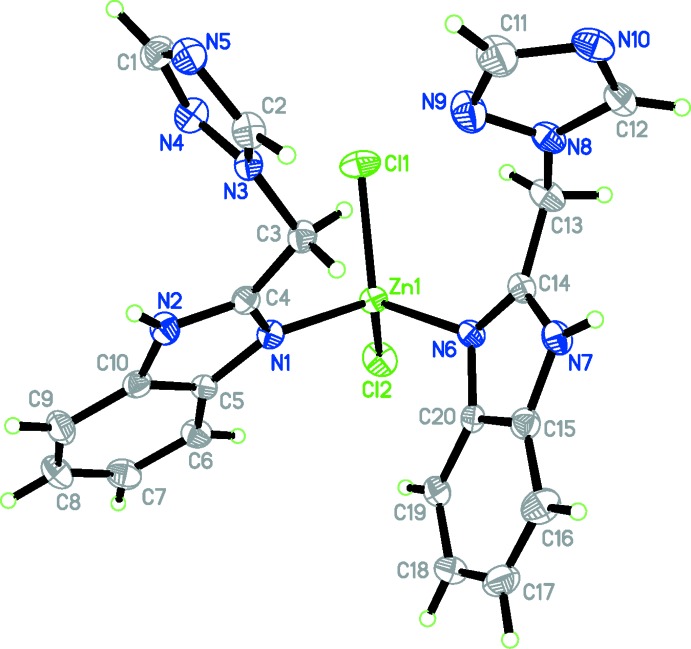
View of the title complex showing 30% probability displacement ellipsolids.

## supplementary crystallographic information

### Comment

In recent years we have focused our attention on the design and synthesis of complexes based on the 2-[(1*H*-1,2,4-triazol-1-yl)methyl]-1*H*-benzimidazole (tmb) ligand since it possesses various coordination modes and can act as both a hydrogen bond acceptor and donor due to the amino group of benzimidazole ring and N atoms of benzimidazole and imidazole rings (Jin *et al.*, 2012; Wang *et al.*, 2012). In order to enrich the categories and numbers of complexes with this ligand, we have selected tmb as the ligand to self-assemble with ZnCl_2_. The crystal structure of the title complex is reported herein.

As shown in Figure 1, two 2-((1*H*-1,2,4-triazol-1-yl)methyl)-1*H*-benzimidazole ligands and two Cl ligands coordinate to the Zn^II^ ion resulting in a slightly distorted tetrahedral geometry. In the tmb ligands, the benzimidazole rings systems are essentially planar with maximum deviations for an atom of 0.021 (3) for N1 and 0.030 (3) Å for N7. In the tmb ligands, the mean planes of the benzimidazole rings systems form dihedral angles of 73.2 (2) [C4-C10/N1/N2 and C1/C2/N3-N5] and 83.5 (2)° [C14-C20/N6/N7 and C11/C12/N8-N10] with the triazole rings. In the crystal, N—H···N hydrogen bonds link complex molecules into one-dimensional chains along [010]. In addition, weak C—H···Cl and C—H···N hydrogen bonds complete a three-dimensional network. Two weak intramolecular C—H···Cl hydrogen bonds are also observed.

### Experimental

A mixture of ZnCl_2_ (0.1 mmol), 2-[(1*H*-1,2,4-triazol-1-yl)methyl]-1*H*-benzimidazole (tmb; 0.1 mmol), and water (10 ml) was placed in a 25 ml Teflon-lined stainless steel vessel and heated at 373 K for 72 h, then cooled to room temperature. Colourless crystals were obtained from the filtrate and dried in air.

### Refinement

H atoms were positioned geometrically and refined as riding atoms, with C—H = 0.93 (aromatic) Å and 0.97 (CH_2_) Å, N-H = 0.86 Å. All H atoms were refined with U_iso_(H) = 1.2 U_eq_(C,N).

### Crystal data

**Table:**

[ZnCl_2_(C_10_H_9_N_5_)_2_]	*F*(000) = 1088
*M**_r_* = 534.73	*D*_x_ = 1.552 Mg m^−^^3^
Monoclinic, *P*2_1_/*c*	Mo *K*α radiation, λ = 0.71073 Å
*a* = 11.571 (2) Å	Cell parameters from 4370 reflections
*b* = 14.109 (3) Å	θ = 2.1–27.9°
*c* = 16.357 (6) Å	µ = 1.34 mm^−^^1^
β = 121.03 (2)°	*T* = 293 K
*V* = 2288.2 (10) Å^3^	Prism, colourless
*Z* = 4	0.18 × 0.17 × 0.08 mm

### Data collection

**Table:**

Rigaku Saturn diffractometer	4234 independent reflections
Radiation source: fine-focus sealed tube	3396 reflections with *I* > 2σ(*I*)
Graphite monochromator	*R*_int_ = 0.054
Detector resolution: 28.5714 pixels mm^-1^	θ_max_ = 25.5°, θ_min_ = 2.1°
ω scans	*h* = −14→11
Absorption correction: multi-scan (*CrystalClear*; Rigaku/MSC Inc., 2004)	*k* = −16→17
*T*_min_ = 0.795, *T*_max_ = 0.901	*l* = −19→19
15780 measured reflections	

### Refinement

**Table:**

Refinement on *F*^2^	Primary atom site location: structure-invariant direct methods
Least-squares matrix: full	Secondary atom site location: difference Fourier map
*R*[*F*^2^ > 2σ(*F*^2^)] = 0.057	Hydrogen site location: inferred from neighbouring sites
*wR*(*F*^2^) = 0.108	H-atom parameters constrained
*S* = 1.13	*w* = 1/[σ^2^(*F*_o_^2^) + (0.0318*P*)^2^ + 1.7955*P*] where *P* = (*F*_o_^2^ + 2*F*_c_^2^)/3
4234 reflections	(Δ/σ)_max_ < 0.001
298 parameters	Δρ_max_ = 0.29 e Å^−^^3^
0 restraints	Δρ_min_ = −0.37 e Å^−^^3^

### Special details

**Table:**

Geometry. All e.s.d.'s (except the e.s.d. in the dihedral angle between two l.s. planes) are estimated using the full covariance matrix. The cell e.s.d.'s are taken into account individually in the estimation of e.s.d.'s in distances, angles and torsion angles; correlations between e.s.d.'s in cell parameters are only used when they are defined by crystal symmetry. An approximate (isotropic) treatment of cell e.s.d.'s is used for estimating e.s.d.'s involving l.s. planes.
Refinement. Refinement of *F*^2^ against ALL reflections. The weighted *R*-factor *wR* and goodness of fit *S* are based on *F*^2^, conventional *R*-factors *R* are based on *F*, with *F* set to zero for negative *F*^2^. The threshold expression of *F*^2^ > σ(*F*^2^) is used only for calculating *R*-factors(gt) *etc*. and is not relevant to the choice of reflections for refinement. *R*-factors based on *F*^2^ are statistically about twice as large as those based on *F*, and *R*- factors based on ALL data will be even larger.

### Fractional atomic coordinates and isotropic or equivalent isotropic displacement parameters (Å^2^)

**Table:**

	*x*	*y*	*z*	*U*_iso_*/*U*_eq_	
Zn1	0.68322 (4)	0.25039 (3)	0.24838 (3)	0.03071 (14)	
Cl1	0.45848 (10)	0.23530 (8)	0.15278 (7)	0.0509 (3)	
Cl2	0.78345 (11)	0.26118 (8)	0.16349 (8)	0.0497 (3)	
N1	0.7393 (3)	0.1337 (2)	0.3354 (2)	0.0292 (7)	
N2	0.7295 (3)	0.0240 (2)	0.4293 (2)	0.0355 (8)	
H2B	0.7070	−0.0035	0.4660	0.043*	
N3	0.4890 (3)	0.1159 (2)	0.4056 (2)	0.0296 (7)	
N4	0.4098 (3)	0.0541 (2)	0.3346 (2)	0.0397 (8)	
N5	0.3487 (3)	0.0612 (2)	0.4450 (2)	0.0408 (8)	
N6	0.7382 (3)	0.3573 (2)	0.3450 (2)	0.0296 (7)	
N7	0.7435 (3)	0.4386 (2)	0.4625 (2)	0.0364 (8)	
H7B	0.7163	0.4726	0.4931	0.044*	
N8	0.4444 (3)	0.4297 (2)	0.3544 (2)	0.0365 (8)	
N9	0.4108 (4)	0.3434 (3)	0.3729 (3)	0.0538 (10)	
N10	0.3299 (3)	0.4606 (3)	0.4226 (2)	0.0451 (9)	
C1	0.3285 (4)	0.0233 (3)	0.3628 (3)	0.0430 (10)	
H1A	0.2617	−0.0214	0.3284	0.052*	
C2	0.4508 (4)	0.1196 (3)	0.4693 (3)	0.0365 (9)	
H2A	0.4902	0.1579	0.5234	0.044*	
C3	0.5917 (4)	0.1708 (3)	0.4012 (3)	0.0343 (9)	
H3A	0.6427	0.2069	0.4596	0.041*	
H3B	0.5485	0.2153	0.3486	0.041*	
C4	0.6863 (4)	0.1093 (3)	0.3879 (3)	0.0303 (8)	
C5	0.8245 (4)	0.0578 (2)	0.3448 (3)	0.0298 (8)	
C6	0.9044 (4)	0.0445 (3)	0.3054 (3)	0.0394 (10)	
H6A	0.9119	0.0906	0.2677	0.047*	
C7	0.9728 (4)	−0.0410 (3)	0.3253 (3)	0.0488 (11)	
H7A	1.0272	−0.0526	0.2999	0.059*	
C8	0.9624 (4)	−0.1100 (3)	0.3820 (3)	0.0532 (12)	
H8A	1.0091	−0.1667	0.3930	0.064*	
C9	0.8851 (4)	−0.0962 (3)	0.4218 (3)	0.0474 (11)	
H9A	0.8789	−0.1419	0.4603	0.057*	
C10	0.8166 (4)	−0.0113 (3)	0.4024 (3)	0.0332 (9)	
C11	0.3423 (5)	0.3666 (3)	0.4131 (4)	0.0575 (13)	
H11A	0.3046	0.3214	0.4339	0.069*	
C12	0.3953 (4)	0.4976 (3)	0.3841 (3)	0.0391 (10)	
H12A	0.4055	0.5622	0.3784	0.047*	
C13	0.5230 (4)	0.4372 (3)	0.3080 (3)	0.0402 (10)	
H13A	0.5202	0.5021	0.2876	0.048*	
H13B	0.4825	0.3973	0.2516	0.048*	
C14	0.6671 (4)	0.4081 (2)	0.3725 (3)	0.0312 (8)	
C15	0.8726 (4)	0.4064 (3)	0.4977 (3)	0.0357 (9)	
C16	0.9907 (4)	0.4177 (3)	0.5860 (3)	0.0474 (11)	
H16A	0.9913	0.4492	0.6362	0.057*	
C17	1.1066 (4)	0.3798 (3)	0.5951 (3)	0.0501 (11)	
H17A	1.1878	0.3864	0.6526	0.060*	
C18	1.1045 (4)	0.3318 (3)	0.5199 (3)	0.0453 (10)	
H18A	1.1848	0.3076	0.5284	0.054*	
C19	0.9880 (4)	0.3192 (3)	0.4339 (3)	0.0394 (10)	
H19A	0.9879	0.2866	0.3845	0.047*	
C20	0.8700 (4)	0.3568 (2)	0.4231 (3)	0.0303 (8)	

### Atomic displacement parameters (Å^2^)

**Table:**

	*U*^11^	*U*^22^	*U*^33^	*U*^12^	*U*^13^	*U*^23^
Zn1	0.0327 (3)	0.0346 (2)	0.0271 (2)	−0.0017 (2)	0.01704 (19)	0.0012 (2)
Cl1	0.0329 (6)	0.0760 (8)	0.0360 (6)	−0.0080 (5)	0.0122 (5)	0.0010 (5)
Cl2	0.0603 (7)	0.0596 (7)	0.0490 (6)	0.0071 (6)	0.0422 (6)	0.0101 (5)
N1	0.0346 (18)	0.0314 (16)	0.0269 (16)	−0.0019 (14)	0.0196 (15)	0.0015 (13)
N2	0.042 (2)	0.0355 (18)	0.0383 (19)	0.0013 (15)	0.0270 (17)	0.0085 (15)
N3	0.0360 (18)	0.0282 (16)	0.0311 (17)	0.0003 (14)	0.0219 (15)	0.0011 (13)
N4	0.046 (2)	0.043 (2)	0.0349 (19)	−0.0053 (16)	0.0247 (17)	−0.0046 (16)
N5	0.046 (2)	0.042 (2)	0.048 (2)	0.0040 (17)	0.0343 (18)	0.0073 (17)
N6	0.0311 (17)	0.0321 (17)	0.0295 (17)	0.0040 (13)	0.0182 (15)	0.0021 (14)
N7	0.043 (2)	0.0366 (18)	0.0391 (19)	0.0059 (15)	0.0285 (17)	−0.0036 (15)
N8	0.0377 (19)	0.043 (2)	0.0364 (19)	0.0090 (15)	0.0249 (16)	0.0064 (15)
N9	0.068 (3)	0.046 (2)	0.073 (3)	0.0085 (19)	0.054 (2)	0.008 (2)
N10	0.040 (2)	0.062 (2)	0.040 (2)	0.0096 (18)	0.0247 (18)	0.0006 (18)
C1	0.041 (2)	0.042 (2)	0.046 (3)	−0.006 (2)	0.023 (2)	0.002 (2)
C2	0.048 (3)	0.037 (2)	0.036 (2)	0.0027 (19)	0.029 (2)	0.0005 (18)
C3	0.041 (2)	0.031 (2)	0.041 (2)	−0.0027 (17)	0.028 (2)	0.0004 (17)
C4	0.031 (2)	0.031 (2)	0.028 (2)	−0.0020 (16)	0.0151 (17)	−0.0018 (16)
C5	0.026 (2)	0.030 (2)	0.031 (2)	0.0007 (16)	0.0129 (17)	−0.0004 (16)
C6	0.037 (2)	0.046 (2)	0.041 (2)	0.0018 (19)	0.024 (2)	0.0052 (19)
C7	0.042 (3)	0.057 (3)	0.055 (3)	0.007 (2)	0.030 (2)	−0.001 (2)
C8	0.047 (3)	0.043 (3)	0.068 (3)	0.016 (2)	0.029 (3)	0.004 (2)
C9	0.047 (3)	0.036 (2)	0.064 (3)	0.010 (2)	0.032 (2)	0.012 (2)
C10	0.030 (2)	0.036 (2)	0.036 (2)	−0.0032 (17)	0.0180 (18)	0.0007 (17)
C11	0.064 (3)	0.057 (3)	0.075 (4)	0.006 (2)	0.052 (3)	0.011 (3)
C12	0.039 (2)	0.041 (2)	0.036 (2)	0.0101 (19)	0.018 (2)	0.0000 (18)
C13	0.042 (2)	0.052 (3)	0.035 (2)	0.014 (2)	0.025 (2)	0.0104 (19)
C14	0.036 (2)	0.030 (2)	0.034 (2)	0.0039 (17)	0.0231 (19)	0.0039 (17)
C15	0.037 (2)	0.034 (2)	0.039 (2)	−0.0007 (17)	0.0214 (19)	−0.0002 (18)
C16	0.048 (3)	0.057 (3)	0.036 (2)	−0.008 (2)	0.021 (2)	−0.007 (2)
C17	0.039 (3)	0.051 (3)	0.044 (3)	−0.009 (2)	0.010 (2)	0.001 (2)
C18	0.031 (2)	0.044 (2)	0.054 (3)	0.0043 (19)	0.017 (2)	0.003 (2)
C19	0.037 (2)	0.032 (2)	0.049 (3)	0.0040 (18)	0.023 (2)	−0.0036 (19)
C20	0.033 (2)	0.0229 (18)	0.035 (2)	−0.0013 (15)	0.0177 (18)	−0.0009 (16)

### Geometric parameters (Å, º)

**Table:**

Zn1—N6	2.035 (3)	C3—C4	1.498 (5)
Zn1—N1	2.051 (3)	C3—H3A	0.9700
Zn1—Cl2	2.2264 (11)	C3—H3B	0.9700
Zn1—Cl1	2.2496 (13)	C5—C6	1.385 (5)
N1—C4	1.332 (4)	C5—C10	1.392 (5)
N1—C5	1.410 (4)	C6—C7	1.386 (5)
N2—C4	1.344 (4)	C6—H6A	0.9300
N2—C10	1.384 (4)	C7—C8	1.393 (6)
N2—H2B	0.8601	C7—H7A	0.9300
N3—C2	1.326 (4)	C8—C9	1.364 (6)
N3—N4	1.361 (4)	C8—H8A	0.9300
N3—C3	1.451 (4)	C9—C10	1.379 (5)
N4—C1	1.315 (5)	C9—H9A	0.9300
N5—C2	1.323 (5)	C11—H11A	0.9300
N5—C1	1.352 (5)	C12—H12A	0.9300
N6—C14	1.333 (4)	C13—C14	1.500 (5)
N6—C20	1.396 (5)	C13—H13A	0.9700
N7—C14	1.339 (5)	C13—H13B	0.9700
N7—C15	1.372 (5)	C15—C16	1.394 (6)
N7—H7B	0.8600	C15—C20	1.394 (5)
N8—C12	1.327 (5)	C16—C17	1.379 (6)
N8—N9	1.358 (4)	C16—H16A	0.9300
N8—C13	1.458 (4)	C17—C18	1.394 (6)
N9—C11	1.306 (5)	C17—H17A	0.9300
N10—C12	1.317 (5)	C18—C19	1.368 (6)
N10—C11	1.351 (5)	C18—H18A	0.9300
C1—H1A	0.9300	C19—C20	1.389 (5)
C2—H2A	0.9300	C19—H19A	0.9300
			
N6—Zn1—N1	101.28 (12)	C5—C6—H6A	121.8
N6—Zn1—Cl2	112.27 (9)	C7—C6—H6A	121.8
N1—Zn1—Cl2	114.06 (9)	C6—C7—C8	122.0 (4)
N6—Zn1—Cl1	113.37 (9)	C6—C7—H7A	119.0
N1—Zn1—Cl1	104.20 (9)	C8—C7—H7A	119.0
Cl2—Zn1—Cl1	111.11 (5)	C9—C8—C7	121.4 (4)
C4—N1—C5	105.2 (3)	C9—C8—H8A	119.3
C4—N1—Zn1	124.7 (2)	C7—C8—H8A	119.3
C5—N1—Zn1	129.7 (2)	C8—C9—C10	117.1 (4)
C4—N2—C10	107.7 (3)	C8—C9—H9A	121.5
C4—N2—H2B	126.1	C10—C9—H9A	121.5
C10—N2—H2B	126.2	C9—C10—N2	131.8 (4)
C2—N3—N4	110.2 (3)	C9—C10—C5	122.2 (4)
C2—N3—C3	129.2 (3)	N2—C10—C5	106.0 (3)
N4—N3—C3	120.5 (3)	N9—C11—N10	115.5 (4)
C1—N4—N3	101.6 (3)	N9—C11—H11A	122.2
C2—N5—C1	102.3 (3)	N10—C11—H11A	122.2
C14—N6—C20	105.5 (3)	N10—C12—N8	110.4 (4)
C14—N6—Zn1	131.3 (3)	N10—C12—H12A	124.8
C20—N6—Zn1	117.6 (2)	N8—C12—H12A	124.8
C14—N7—C15	108.3 (3)	N8—C13—C14	112.3 (3)
C14—N7—H7B	125.9	N8—C13—H13A	109.1
C15—N7—H7B	125.8	C14—C13—H13A	109.1
C12—N8—N9	109.9 (3)	N8—C13—H13B	109.1
C12—N8—C13	129.6 (3)	C14—C13—H13B	109.1
N9—N8—C13	120.5 (3)	H13A—C13—H13B	107.9
C11—N9—N8	101.9 (3)	N6—C14—N7	111.8 (3)
C12—N10—C11	102.3 (3)	N6—C14—C13	124.4 (3)
N4—C1—N5	115.5 (4)	N7—C14—C13	123.4 (3)
N4—C1—H1A	122.2	N7—C15—C16	132.1 (4)
N5—C1—H1A	122.2	N7—C15—C20	105.7 (3)
N5—C2—N3	110.3 (3)	C16—C15—C20	122.1 (4)
N5—C2—H2A	124.8	C17—C16—C15	116.6 (4)
N3—C2—H2A	124.8	C17—C16—H16A	121.7
N3—C3—C4	112.1 (3)	C15—C16—H16A	121.7
N3—C3—H3A	109.2	C16—C17—C18	121.3 (4)
C4—C3—H3A	109.2	C16—C17—H17A	119.3
N3—C3—H3B	109.2	C18—C17—H17A	119.3
C4—C3—H3B	109.2	C19—C18—C17	122.0 (4)
H3A—C3—H3B	107.9	C19—C18—H18A	119.0
N1—C4—N2	112.5 (3)	C17—C18—H18A	119.0
N1—C4—C3	123.9 (3)	C18—C19—C20	117.7 (4)
N2—C4—C3	123.7 (3)	C18—C19—H19A	121.2
C6—C5—C10	120.9 (3)	C20—C19—H19A	121.2
C6—C5—N1	130.5 (3)	C19—C20—C15	120.3 (4)
C10—C5—N1	108.6 (3)	C19—C20—N6	131.1 (3)
C5—C6—C7	116.5 (4)	C15—C20—N6	108.6 (3)
			
N6—Zn1—N1—C4	63.8 (3)	C8—C9—C10—N2	178.7 (4)
Cl2—Zn1—N1—C4	−175.4 (3)	C8—C9—C10—C5	0.2 (6)
Cl1—Zn1—N1—C4	−54.1 (3)	C4—N2—C10—C9	−177.6 (4)
N6—Zn1—N1—C5	−125.0 (3)	C4—N2—C10—C5	1.1 (4)
Cl2—Zn1—N1—C5	−4.2 (3)	C6—C5—C10—C9	−1.2 (6)
Cl1—Zn1—N1—C5	117.1 (3)	N1—C5—C10—C9	177.5 (4)
C2—N3—N4—C1	−0.9 (4)	C6—C5—C10—N2	180.0 (3)
C3—N3—N4—C1	−177.1 (3)	N1—C5—C10—N2	−1.4 (4)
N1—Zn1—N6—C14	−97.6 (3)	N8—N9—C11—N10	−0.4 (6)
Cl2—Zn1—N6—C14	140.3 (3)	C12—N10—C11—N9	0.6 (6)
Cl1—Zn1—N6—C14	13.4 (3)	C11—N10—C12—N8	−0.5 (5)
N1—Zn1—N6—C20	51.9 (3)	N9—N8—C12—N10	0.3 (5)
Cl2—Zn1—N6—C20	−70.1 (3)	C13—N8—C12—N10	−179.9 (4)
Cl1—Zn1—N6—C20	163.0 (2)	C12—N8—C13—C14	108.7 (4)
C12—N8—N9—C11	0.0 (5)	N9—N8—C13—C14	−71.5 (5)
C13—N8—N9—C11	−179.8 (4)	C20—N6—C14—N7	−0.9 (4)
N3—N4—C1—N5	0.7 (5)	Zn1—N6—C14—N7	151.4 (3)
C2—N5—C1—N4	−0.2 (5)	C20—N6—C14—C13	172.7 (3)
C1—N5—C2—N3	−0.4 (4)	Zn1—N6—C14—C13	−35.1 (5)
N4—N3—C2—N5	0.9 (4)	C15—N7—C14—N6	−0.3 (4)
C3—N3—C2—N5	176.7 (3)	C15—N7—C14—C13	−174.0 (3)
C2—N3—C3—C4	130.2 (4)	N8—C13—C14—N6	139.7 (4)
N4—N3—C3—C4	−54.4 (4)	N8—C13—C14—N7	−47.5 (5)
C5—N1—C4—N2	−0.5 (4)	C14—N7—C15—C16	179.6 (4)
Zn1—N1—C4—N2	172.5 (2)	C14—N7—C15—C20	1.4 (4)
C5—N1—C4—C3	178.2 (3)	N7—C15—C16—C17	−176.0 (4)
Zn1—N1—C4—C3	−8.8 (5)	C20—C15—C16—C17	2.0 (6)
C10—N2—C4—N1	−0.4 (4)	C15—C16—C17—C18	−0.7 (6)
C10—N2—C4—C3	−179.1 (3)	C16—C17—C18—C19	−0.5 (7)
N3—C3—C4—N1	143.9 (3)	C17—C18—C19—C20	0.5 (6)
N3—C3—C4—N2	−37.6 (5)	C18—C19—C20—C15	0.7 (6)
C4—N1—C5—C6	179.6 (4)	C18—C19—C20—N6	178.6 (4)
Zn1—N1—C5—C6	7.1 (6)	N7—C15—C20—C19	176.4 (3)
C4—N1—C5—C10	1.2 (4)	C16—C15—C20—C19	−2.1 (6)
Zn1—N1—C5—C10	−171.4 (2)	N7—C15—C20—N6	−1.9 (4)
C10—C5—C6—C7	1.1 (6)	C16—C15—C20—N6	179.7 (3)
N1—C5—C6—C7	−177.2 (4)	C14—N6—C20—C19	−176.3 (4)
C5—C6—C7—C8	−0.2 (6)	Zn1—N6—C20—C19	26.9 (5)
C6—C7—C8—C9	−0.7 (7)	C14—N6—C20—C15	1.7 (4)
C7—C8—C9—C10	0.7 (7)	Zn1—N6—C20—C15	−155.0 (2)

### Hydrogen-bond geometry (Å, º)

**Table:**

*D*—H···*A*	*D*—H	H···*A*	*D*···*A*	*D*—H···*A*
N2—H2*B*···N5^i^	0.86	2.04	2.899 (4)	177
N7—H7*B*···N10^ii^	0.86	1.96	2.814 (4)	172
C3—H3*B*···Cl1	0.97	2.83	3.641 (4)	142
C13—H13*B*···Cl1	0.97	2.73	3.628 (4)	154
C2—H2*A*···Cl1^iii^	0.93	2.77	3.596 (4)	148
C13—H13*A*···N4^iv^	0.97	2.62	3.261 (5)	124
C18—H18*A*···Cl1^v^	0.93	2.81	3.635 (4)	149

Symmetry codes: (i) −*x*+1, −*y*, −*z*+1; (ii) −*x*+1, −*y*+1, −*z*+1; (iii) *x*, −*y*+1/2, *z*+1/2; (iv) −*x*+1, *y*+1/2, −*z*+1/2; (v) *x*+1, −*y*+1/2, *z*+1/2.
